# Assessing the efficiency of eligibility criteria for low-dose computed tomography lung screening in China according to current guidelines

**DOI:** 10.1186/s12916-024-03445-5

**Published:** 2024-06-26

**Authors:** Yanyan Tang, Lina Zhou, Fei Wang, Yao Huang, Jianwei Wang, Shijun Zhao, Linlin Qi, Li Liu, Min Liang, Donghui Hou, Zhijian Xu, Kai Zhang, Wei Tang, Ning Wu

**Affiliations:** 1 Department of Diagnostic Radiology, National Cancer Center/National Clinical Research Center for Cancer/Cancer Hospital, Chinese Academy of Medical Sciences and Peking Union Medical College, Beijing, 100021 China; 2 Office of Cancer Screening, National Cancer Center/National Clinical Research Center for Cancer/Cancer Hospital, Chinese Academy of Medical Sciences and Peking Union Medical College, Beijing, 100021 China; 3 Department of Diagnostic Radiology, Beijing Chaoyang Hospital, Capital Medical University, Beijing, 100020 China; 4 Department of Cancer Prevention, National Cancer Center/National Clinical Research Center for Cancer/Cancer Hospital, Chinese Academy of Medical Sciences and Peking Union Medical College, Beijing, 100020 China; 5 Department of Nuclear Medicine (PET-CT Center), National Cancer Center/National Clinical Research Center for Cancer/Cancer Hospital, Chinese Academy of Medical Sciences and Peking Union Medical College, Beijing, 100021 China

**Keywords:** Lung cancer screening, LDCT, Guideline, Eligibility criteria, Efficiency

## Abstract

**Background:**

Evidence from observational studies indicates that lung cancer screening (LCS) guidelines with high rates of lung cancer (LC) underdiagnosis, and although current screening guidelines have been updated and eligibility criteria for screening have been expanded, there are no studies comparing the efficiency of LCS guidelines in Chinese population.

**Methods:**

Between 2005 and 2022, 31,394 asymptomatic individuals were screened using low-dose computed tomography (LDCT) at our institution. Demographic data and relevant LC risk factors were collected. The efficiency of the LCS for each guideline criteria was expressed as the efficiency ratio (ER). The inclusion rates, eligibility rates, LC detection rates, and ER based on the different eligibility criteria of the four guidelines were comparatively analyzed. The four guidelines were as follows: China guideline for the screening and early detection of lung cancer (CGSL), the National Comprehensive Cancer Network (NCCN), the United States Preventive Services Task Force (USPSTF), and International Early Lung Cancer Action Program (I-ELCAP).

**Results:**

Of 31,394 participants, 298 (155 women, 143 men) were diagnosed with LC. For CGSL, NCCN, USPSTF, and I-ELCAP guidelines, the eligibility rates for guidelines were 13.92%, 6.97%, 6.81%, and 53.46%; ERe for eligibility criteria were 1.46%, 1.64%, 1.51%, and 1.13%, respectively; and for the inclusion rates, they were 19.0%, 9.5%, 9.3%, and 73.0%, respectively. LCs which met the screening criteria of CGSL, NCCN, USPSTF, and I-ELCAP guidelines were 29.2%, 16.4%, 14.8%, and 86.6%, respectively. The age and smoking criteria for CGSL were stricter, hence resulting in lower rates of LC meeting the screening criteria. The CGSL, NCCN, and USPSTF guidelines showed the highest underdiagnosis in the 45–49 age group (17.4%), while the I-ELCAP guideline displayed the highest missed diagnosis rate (3.0%) in the 35–39 age group. Males and females significantly differed in eligibility based on the criteria of the four guidelines (*P* < 0.001).

**Conclusions:**

The I-ELCAP guideline has the highest eligibility rate for both males and females. But its actual efficiency ratio for those deemed eligible by the guideline was the lowest. Whereas the NCCN guideline has the highest ERe value for those deemed eligible by the guideline.

**Supplementary Information:**

The online version contains supplementary material available at 10.1186/s12916-024-03445-5.

## Background

According to Global Cancer Statistics 2020, lung cancer is the most common cause of morbidity and mortality in men and the third leading type of cancer in women, resulting in a high socioeconomic burden [1]. Among the malignant tumors, lung cancer has the highest incidence and mortality rates in China [2]. Studies have demonstrated that the 5-year survival rate for lung cancer decreases as the stage of diagnosis increases; the overall 5-year survival rate for lung cancer decreases from over 80% for stage I to < 10% for stage IV and even 0 for stage IVB [3]. Therefore, early diagnosis and timely treatment are the most effective methods to reduce lung cancer mortality. Low-dose computed tomography (LDCT) is effective for detecting lung cancer at an early stage. The National Lung Screening Trial (NLST) demonstrated for the first time in 2011 that LDCT screening substantially reduced lung cancer mortality [4]. The results from the NELSON lung cancer screening (LCS) study also confirmed the benefits of LDCT in reducing lung cancer mortality [5]. In addition, a multicenter prospective cohort study at the National Cancer Center demonstrated that lung cancer mortality was 31.0% lower, and all-cause mortality was 32.0% lower in the screened group than in the non-screened group [6]. Therefore, LDCT is used worldwide as the main screening method for people at a high risk of lung cancer.

In theory, 88% of lung cancer deaths could be avoided if screening accurately targeted all high-risk participants [7]. However, screening for lung cancer (LC) using LDCT is associated with some inherent risks, such as increased radiation risk, unnecessary invasive procedures, and economic burden. Therefore, to ensure benefits to the screened population, LCS in high-risk groups is recommended by guidelines or consensuses published by countries worldwide. Rational and accurate selection of screened individuals can reduce the proportion of ineffective screening and improve the health economics of LCS. The current national and international LCS guidelines differ in their definitions of screening populations. The eligibility criteria in guidelines are mainly based on epidemiological risk factors, such as age, number of pack-years smoked, and length of time since smoking cessation. The National Cancer Center of China (NCC) issued guidelines for LCS, entitled *China guideline for the screening and early detection of lung cancer* (CGSL) (2021, Beijing) [8], which considered risk factors other than smoking and age. However, this guideline-based screening protocol lacks a large population-based real-world study to determine whether it is more suitable than foreign screening protocols for LCS in China. Several studies have evaluated the use of updated versions of guideline eligibility criteria in LCS. However, these studies have looked at Europeans and Americans [9, 10], and there is limited information on updated criteria such as USPSTF2021 and NCCN2022 regarding Asians, especially Chinese. Although some LCS have been conducted in China about eligibility criteria, the eligibility criteria were applied from pre-update screening guidelines, such as the NLST or the USPSTF2013 [11, 12]. A recent cross-sectional study, although using the CGSL guideline criteria to characterize a population in China, did not assess the efficiency of the eligibility criteria or compare them with foreign guidelines [13]. Thus, the CGSL guideline lacks real-world application and comparative studies in Chinese population.

Whether the implementation of the CGSL screening criteria in the Chinese population is more efficient than that of other guidelines remains an important question. Therefore, this study aimed to compare the efficiency of four major national and international guidelines on LCS eligibility: CGSL, National Comprehensive Cancer Network (NCCN), USPSTF,and International Early Lung Cancer Action Program (I-ELCAP).

## Methods

### Study design and population

This retrospective study was approved by the Ethics Committee of the NCC/Cancer Hospital of the Chinese Academy of Medical Sciences (No.14–115/905). The approval allowed participants or their institutions to pay for the LCS.

Between July 2005 and June 2022, 42,856 individuals who enrolled in the LCS registry completed questionnaires at the Department of Cancer Prevention of the Cancer Hospital, Chinese Academy of Medical Sciences, and NCC. We defined the target population as asymptomatic individuals enrolled in the Lung Cancer Screening Registry. The initial cohort of participants had volunteered for LCS on their own and paid for LDCT screening by themselves or their institutions. Participants were excluded if they met any of the following criteria: medical or psychiatric problems that prevented them from completing informed consent and LDCT; current medical conditions that prevented them from undergoing invasive procedures, such as biopsy, puncture, and surgery required to receive a positive result; history of or treatment for malignancy (excluding basal cell carcinoma of the skin and carcinoma in situ of the cervix); previous surgical removal of lung tissue (including whole lung, lobes, segments, wedges, excluding lung puncture and lung biopsy); symptoms of lung cancer (such as unexplained weight loss or coughing of blood); and certain life-threatening diseases, such as severe cardiovascular disease, severe kidney disease, and liver cirrhosis. In addition, the participants were required to sign an informed consent form and complete a questionnaire to make an appointment for LDCT. Basic demographic data, lung cancer risk factors, family history, and baseline comorbidities were recorded using questionnaires.

The basic demographic data included age and sex, and lung cancer risk factors included smoking status, passive smoking, history of occupational exposure, family history of lung cancer, and chronic obstructive pulmonary disease (COPD). Smokers were defined as former or current smokers who smoked more than once a day for at least 1 year. Age at the start of regular smoking and years of smoking were recorded (years since quitting and previous smoking status were filled in if they had quit smoking). Cumulative smoking was expressed using the smoking index (SI), where SI (pack-years) = number of packs smoked per day × number of years smoked. Passive smoking was defined as people exposed to secondhand smoke for more than 20 years by living or working in the same room with smokers. History of occupational exposure was defined as occupational exposure to asbestos or soot for > 1 year.

### Imaging analysis and follow-up procedures

The pulmonary nodule management program and follow-up modalities in this study were based on the I-ELCAP protocol, with detailed information referenced in our published article, which provides detailed information on LDCT parameters, nodule imaging assessment, and management [14]. Two research assistants performed diagnostic assessments and data recording of associated complications for lung nodules identified during screening. Pathology reports and surgery and treatment records were recorded. The World Health Organization (WHO) 2015 classification of lung cancer [15] and the eighth edition of the TNM classification for lung cancer were used for histopathological assessment and tumor staging [3].

### Guidelines eligibility criteria

Table 1 describes the four screening criteria used in this study. The CGSL (2021) guideline applies to participants aged 50–74 years who are at high risk of lung cancer and meet at least one of the following criteria: history of smoking (smoking ≥ 30 packs/year); passive smoking (≥ 20 years); COPD; history of occupational exposure (≥ 1 year); and a first-degree relative with a confirmed lung cancer diagnosis. The NCCN (2022) recommends LDCT screening for individuals aged ≥ 50 years with a history of ≥ 20 pack-years of smoking [16]. The I-ELCAP (2006) criteria are as follows: age ≥ 40 years; current or former smoker; exposure to secondhand smoke; or occupational exposure to asbestos, beryllium, uranium, or radon [17]. The USPSTF (2021) recommends annual screening using LDCT for adults aged 50–80 years with a 20-pack-year smoking history who are current smokers or who have quit smoking within the past 15 years [18].

**Table 1:** Criteria used by CGSL, NCCN, USPSTF, and I-ELCAP

Screening protocol	Age	History of cigarette smoking	Other
CGSL	50–74 years	Smoking ≥ 30 packs/year (including ever smoked ≥ 30 packs/year, but quit < 15 years); passive smoking ≥ 20 years	Chronic obstructive pulmonary disease; history of occupational exposure ≥ 1 year; having a first-degree relative with a confirmed lung cancer diagnosis
NCCN	≥ 50 years	Smoking ≥ 20 packs/year	N/A
USPSTF	50–80 years	Smoking ≥ 20 packs/year Quit smoking time < 15 years	N/A
I-ELCAP	≥ 40 years	A current or former smoker, exposure to second-hand smoke	Occupational exposure to asbestos, beryllium, uranium, or radon

*Abbreviations*: *N/A* Not applicable, *CGSL* Chinese guidelines for the screening and early detection of lung cancer, *NCCN* National Comprehensive Cancer Network, *USPSTF* US Preventive Services Task Force, *I-ELCAP* International Early Lung Cancer Action Program

### Outcomes of interest

Our study expressed the efficiency of LCS for each guideline criteria as an efficiency ratio (ER), the ERe was defined as the number of lung cancers that met the guideline eligibility criteria divided by those deemed eligible based on the guideline, and the ERi was defined as the number of lung cancers that did not meet the guideline eligibility criteria divided by those deemed ineligible based on the guideline. A higher ERe value indicated a higher detection rate of lung cancer. The eligibility rate was defined as the total number of participants deemed eligible for LCS based on the guideline/the total number of participants enrolled in the registry.

### Statistical analysis

All statistical analyses were performed using the SPSS software (version 25.0; SPSS Inc., Chicago, IL, USA). Continuous variables were expressed as mean ± standard deviation (SD). Differences in continuous variables between the two groups were compared using independent Student’s *t*-tests. Categorical variables were expressed as frequencies and percentages, and Fisher’s exact or chi-square test was used to compare categorical variables and test for differences in baseline characteristics between men and women. Statistical significance was set at *P* < 0.05.

## Results

### Baseline screening results

**Fig. 1:**
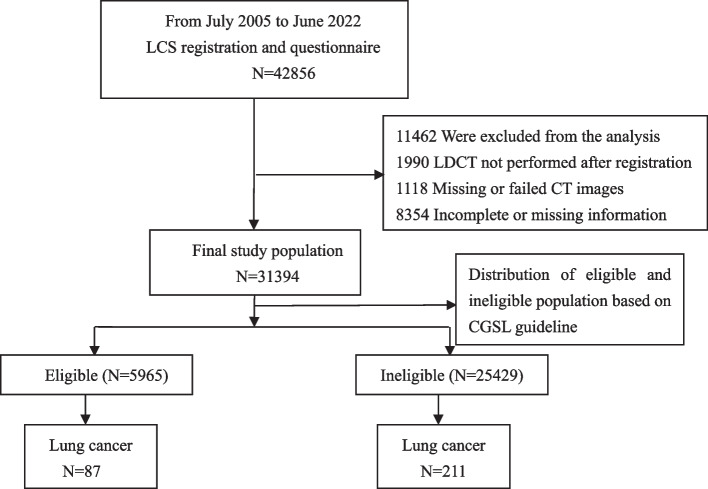
Flow chart of study participants and baseline screening results based on CGSL guideline. Abbreviations: LCS, lung cancer screening; LDCT, low-dose computed tomography; CGSL, China guideline for the screening and early detection of lung cancer

After excluding 11,462 participants from the study population, we retrospectively analyzed 31,394 consecutive asymptomatic participants (17,490 men and 13,904 women). The baseline screening results demonstrated that among the 31,394 asymptomatic participants, 298 (155 women and 143 men) were diagnosed with lung cancer. Among the 31,394 participants, 5965 were eligible for LCS, leaving 25,429 individuals ineligible according to the CGSL criteria (Fig. 1).

### Characteristics of study participants

Table 2 summarizes and compares the baseline demographic and clinical characteristics of the participants (both sexes). The mean ages for men and women were 48.8 ± 10.2 and 49.8 ± 10.1 years, respectively, with an overall mean age of 49.3 ± 10.2 years. Significant differences were observed in baseline characteristics between the sexes (all *P* < 0.001). The percentage of smoking, age, passive smoking, and occupational exposure was higher in men than in women. In contrast, the percentage of family history of lung cancer was significantly higher in women than in men (*P* < 0.001). Additional File: Table S1 demonstrates the characteristics of study participants deemed eligible by the four guidelines for male and female subgroups. The four guidelines differed significantly in age, smoking, passive smoking, history of occupational exposure, family history of lung cancer, and COPD (*P* < 0.001).

**Table 2:** Characteristics of study participants stratified by sex

Characteristic	Total, *n* (%) 31,394 (100)	Male, *n* (%) 17,490 (55.7)	Female, *n* (%) 13,904 (44.3)	*P* value
Age range				＜0.001
~ 39	5127 (16.3)	3163 (18.1)	1964 (14.1)	
40–44	5411 (17.2)	3078 (17.6)	2333 (16.8)	
45–49	6222 (19.8)	3414 (19.5)	2808 (20.2)	
50–54	5410 (17.2)	2899 (16.6)	2511 (18.1)	
55–59	4166 (13.3)	2311 (13.2)	1855 (13.3)	
60–64	2714 (8.6)	1378 (7.9)	1336 (9.6)	
65–69	1430 (4.6)	756 (4.3)	674 (4.8)	
70–74	555 (1.8)	297 (1.7)	258 (1.9)	
75–79	243 (0.8)	134 (0.8)	109 (0.8)	
80 ~	116 (0.4)	60 (0.3)	56 (0.4)	
Mean age ± SD (years)	49.3 ± 10.2	48.8 ± 10.2	49.8 ± 10.1	< 0.001
Smoking status				< 0.001
Non-smoking	21,427 (68.3)	8183 (46.8)	13,244 (95.2)	
Smoking	9967 (31.7)	9307(53.2)	660 (4.8)	
Smoking volume (pack year)				
< 10	2452 (24.6)	2187 (23.5)	265 (40.2)	
10 ~ 19	2474 (24.8)	2324 (25.0)	150 (22.7)	
20 ~ 29	2289 (23.0)	2147 (23.1)	142 (21.5)	
30 ~	2752 (27.6)	2649 (28.5)	103 (15.6)	
Passive smoking (pack year)				< 0.001
Unknown	17,439 (55.6)	6700 (38.3)	10,739 (77.2)	
No	4629 (14.7)	2089 (11.9)	2540 (18.3)	
Yes	9326 (29.7)	8701 (49.7)	625 (4.5)	
< 20	4622 (49.6)	4227 (48.6)	395 (63.2)	
20–29	2128 (22.8)	1994 (22.9)	134 (21.4)	
30 ~	2576 (27.6)	2480 (28.5)	96 (15.4)	
Quit smoking time (years)				< 0.001
No	28,119 (89.6)	14,395 (82.3)	13,724 (98.7)	
Yes	3275 (10.4)	3095 (17.7)	180 (1.3)	
≥ 15	925 (28.2)	886 (28.6)	39 (21.7)	
< 15	2350 (71.8)	2209 (71.4)	141 (78.3)	
Family history of lung cancer				< 0.001
Yes	5060 (16.1)	2624 (15.0)	2436 (17.5)	
No	26,334 (83.9)	14,866 (85.0)	11,468 (82.5)	
History of occupational exposure				< 0.001
Yes	1492 (5.0)	1213 (7.0)	297 (2.1)	
No	29,902 (95.0)	16,277 (93.0)	13,625 (97.9)	

Abbreviation: *SD* Standard deviation

### Sex stratification based on different guideline eligibility criteria

There was a significant difference in the inclusion rate of the male subgroup and the female subgroup in these 4 guidelines (both *P* < 0.001). According to the criteria of the four guidelines, the I-ELCAP had the highest inclusion rate at 73% (22,911/31,394), followed by the CGSL at 19% (5965/31,394). In addition, the inclusion rates of the NCCN and USPSTF guidelines were low at 9.5% (2989/31,394) and 9.3% (2919/31,394), respectively. In our study, the eligibility gap between men and women for the four LCS criteria was evident (all *P* < 0.001), with female participants being significantly less likely than their male counterparts to meet each guideline criteria (Table 3). According to the NCCN and USPSTF guidelines, only 1.0% and 1.2% of women, respectively, were eligible. Based on the I-ELCAP guidelines, among a significantly larger group of screening-eligible individuals, the percentage of eligible men was 4.8% higher than that of eligible women (Table 3).

**Table 3:** Sex stratification based on different guideline eligibility criteria

**Guideline**	**Male, *n* (%)** **17,490 (55.7)**		**Female, *n* (%)** **13,904 (44.3)**		***P* value**	**Total, *n* (%)**	**Total, *n* (%)**
**Eligibility**	**Eligible**	**Ineligible**	**Eligible**	**Ineligible**		**Eligible**	**Ineligible**
CGSL	4044 (23.1)	13,446 (76.9)	1921 (13.8)	11,983 (86.2)	< 0.001	5965 (19.0)	25,429 (81.0)
NCCN	2845 (16.3)	14,645 (83.7)	144 (1.0)	13,760 (99.0)	< 0.001	2989 (9.5)	28,405 (90.5)
USPSTF	2751 (15.7)	14,739 (84.3)	168 (1.2)	13,736 (98.8)	< 0.001	2919 (9.3)	28,475 (90.7)
I-ELCAP	13,139 (75.1)	4351 (42.9)	9772 (70.3)	4132 (29.7)	< 0.001	22,911 (73.0)	8483 (27.0)
*P* value	< 0.001		< 0.001			< 0.001	

*Abbreviations*: *CGSL* Chinese guidelines for the screening and early detection of lung cancer, *NCCN* National Comprehensive Cancer Network, *USPSTF* US Preventive Services Task Force, *I-ELCAP* International Early Lung Cancer Action Program

### Distribution of lung cancer based on different guideline criteria

Table 4 presents the distribution of lung cancer cases for the various criteria according to different guidelines. According to the CGSL criteria, 29.2% of lung cancer cases met the criteria, and the rates of meeting the requirements for age, smoking, passive smoking, occupational exposure, family history of lung cancer, and COPD were 69.5%, 11.4%, 12.1%, 2.7%, 13.4%, and 8.4%, respectively. Moreover, 70.8% (211/298) of lung cancer cases did not meet these criteria. The rates of meeting the age requirements for the NCCN, USPSTF, and I-ELCAP guidelines were 71.8%, 71.8%, and 96.3%, respectively, and those of meeting the smoking criteria were 19.8%, 14.1%, and 27.5%, respectively. Lung cancer detection rates between the NCCN and USPSTF guideline-eligible participants were 16.4% (49/298) and 14.8% (44/298), respectively. The rates of missed diagnosis for lung cancer were high, at 83.6% (249/298) and 85.2% (254/298), respectively. Compared with other guidelines mentioned above, the I-ELCAP guideline was the only one in which the number of participants with confirmed lung cancer was greater than the number of lung cancers missed, with 258 confirmed lung cancer cases and a lung cancer miss rate of only 13.4% (40/298).

**Table 4:** Distribution of individuals with lung cancer based on different guideline criteria

**Guideline criteria**	**CGSL** ***n* (%)**	**NCCN** ***n* (%)**	**USPSTF** ***n* (%)**	**I-ELCAP** ***n* (%)**
Age				
Met criteria	207 (69.5)	214 (71.8)	214 (71.8)	287 (96.3)
Not met criteria	91 (30.5)	84 (28.2)	84 (28.2)	11 (3.7)
Pack years				
Met criteria	34 (11.4)	59 (19.8)	42 (14.1)	82 (27.5)
Not met criteria	264 (88.6)	239 (80.2)	256 (85.9)	216 (72.5)
Passive smoking				
Met criteria	36 (12.1)	N/A	N/A	251 (84.2)
Not met criteria	262 (87.9)	N/A	N/A	47 (15.8)
Time since quit				
Met criteria	12 (4.0)	N/A	12 (4.0)	N/A
Not met criteria	286 (96.0)	N/A	286 (96.0)	N/A
Occupational exposure				
Met criteria	8 (2.7)	N/A	N/A	8 (2.7)
Not met criteria	290 (97.3)	N/A	N/A	290 (97.3)
Family history of lung cancer				
Met criteria	40 (13.4)	N/A	N/A	N/A
Not met criteria	258 (86.6)	N/A	N/A	N/A
Having COPD				
Met criteria	25 (8.4)	N/A	N/A	N/A
Not met criteria	273 (91.6)	N/A	N/A	N/A
Total Met	87 (29.2)	49 (16.4)	44 (14.8)	258 (86.6)

*Abbreviations*: *COPD* Chronic obstructive pulmonary disease, *N/A* Not applicable, *CGSL* Chinese guidelines for the screening and early detection of lung cancer, *NCCN* National Comprehensive Cancer Network, *USPSTF* US Preventive Services Task Force, *I-ELCAP* International Early Lung Cancer Action Program

### Guideline-based eligibility rates

There was a significant difference between the four guidelines in terms of male, female, and overall eligibility rates (*P* < 0.001), with the I-ELCAP guideline having a significantly higher eligibility rate than the other three guidelines, with male, female, and overall eligibility rates of over 50%, while the NCCN guideline had the lowest eligibility rate, with an eligibility rate of only 0.74% for women (Table 5).

**Table 5:** Guideline-based eligibility rates

**Guideline criteria**	**Male, % (*n*)**	**Female, % (*n*)**	**Total, % (*n*)**
CGSL eligible	17.23 (4044/23,476)	9.91 (1921/19,380)	13.92 (5965/42,856)
NCCN eligible	12.12 (2845/23,476)	0.74 (144/19,380)	6.97 (2989/42,856)
USPSTF eligible	11.72 (2751/23,476)	0.87 (168/19,380)	6.81 (2919/42,856)
I-ELCAP eligible	55.97 (13,139/23,476)	50.42 (9772/19,380)	53.46 (22,911/42,856)
*P* value	< 0.0001	< 0.0001	< 0.0001

### Guideline-based sex stratification of eligibility criteria for lung cancer detection

We observed a wide range of lung cancer detection rates, from 14.8% in the USPSTF guideline to 86.6% in the I-ELCAP guideline. When analyzing the four guidelines, we found significant sex differences in lung cancer diagnosis in all except for the I-ELCAP guideline, with more men than women diagnosed with lung cancer in the guideline-eligible population (*P* < 0.001). In the population ineligible for the USPSTF guideline, women were more likely to miss lung cancer diagnosis than men, with a missed diagnosis rate of 98.7% (153/155) in women (Additional File: Table S2).

### Guideline-based lung cancer detection efficiency

Of all individuals with confirmed lung cancer, 1.11% of 13,904 were women, and 0.82% of 17,490 were men. For the male subgroup and the total population, there were significant differences between the four guidelines in terms of the efficiency with which eligible persons were recognized under the guidelines (*P* < 0.05), whereas there were no differences for the male subgroup (*P* > 0.05). The NCCN guideline had an ERe of 1.64%, which was the most efficient of the four guidelines for detecting lung cancer, and the USPSTF guidelines had the second-highest efficiency at 1.51%. The CGSL guideline, with an ERe of 1.46%, was in third place. Compared with the other three guidelines, the I-ELCAP guideline had the lowest ERe value (1.13%) despite covering the largest number of LCS-eligible participants. Among the LCS-eligible participants, we found no significant differences in the efficiency between men and women across the four guidelines (all *P* > 0.05). However, among the LCS-ineligible participants, except for the I-ELCAP guideline, the efficiencies of the other three guidelines were significantly different between men and women, with females having greater ERi values than their male counterparts (*P* < 0.05) (Table 6).

**Table 6:** Guideline-based lung cancer detection efficiency

**Guideline criteria**	**Male, % (*n*)**		**Female, % (*n*)**		P^*^ value	**Total, ERe, % (*n*)**	**Total, ERi, % (*n*)**
	**Eligible (ERe)**	**Ineligible (ERi)**	**Eligible (ERe)**	**Ineligible (ERi)**			
CGSL	1.41 (57/4044)	0.64 (86/13,446)	1.56 (30/1921)	1.04 (125/11,983)	0.0005	1.46 (87/5965)	0.83 (211/25,429)
NCCN	1.62 (46/2845)	0.66 (97/14,645)	2.08 (3/144)	1.10 (152/13,760)	< 0.0001	1.64 (49/2989)	0.88 (249/28,405)
USPSTF	1.53 (42/2751)	0.69 (101/14,739)	1.19 (2/168)	1.11 (153/13,736)	0.0001	1.51 (44/2919)	0.89 (254/28,475)
I-ELCAP	0.97 (127/13,139)	0.37 (16/4351)	1.34 (131/9772)	0.58 (24/4132)	0.1575	1.13 (258/22,911)	0.47 (40/8483)
*P* value	0.03	0.128	0.664	0.021		0.018	0.02

^*^Male ineligible versus female ineligible

### Lung cancer characteristics based on different guideline eligibility criteria

To compare the characteristics of those participants with lung cancer deemed eligible or ineligible based on the guidelines, we explored risk factors for lung cancer by eligibility criteria. Previous studies from China have shown that lung cancer patients are increasingly occurring at younger ages [19, 20]. To explore the association between lung cancer missed or diagnosed according to the four guidelines and different age groups, we further analyzed the age distribution of lung cancer patients. Those lung cancer patients who were deemed eligible based on the four guidelines showed statistically significant differences in age, smoking, passive smoking, history of occupational exposure, COPD, and family histories of lung cancer risk factors (*P* < 0.05). Those participants who met the CGSL, NCCN, USPSTF, and I-ELCAP guidelines eligibility criteria had the highest probability of being diagnosed with lung cancer in the 55–59 age group, 9.1%, 5.4%, 5.0%, and 18.5%, respectively. The CGSL, NCCN, and USPSTF guidelines showed the highest underdiagnosis in the 45–49 age group (17.4%), while the I-ELCAP guideline displayed the highest missed diagnosis rate (3.0%) in the 35–39 age group. Those deemed eligible based on the four guidelines for lung cancer differed in lung cancer risk factors (*P* < 0.05). The guideline with the highest rates of smokers, passive smokers, family history of lung cancer, and COPD was I-ELCAP, and the highest rates of occupational exposure history were CGSL and I-ELCAP guidelines (Table 7).

**Table 7:** Lung cancer characteristics based on different guideline eligibility criteria

**Characteristics**	**CGSL eligible** ***n* = 87**	**NCCN eligible** ***n* = 49**	**USPSTF eligible** ***n* = 44**	**I-ELCAP eligible** ***n* = 258**	***P*** ^*^ value	**CGSL ineligible** ***n* = 211**	**NCCN ineligible** ***n* = 249**	**USPSTF ineligible** ***n* = 254**	**I-ELCAP ineligible** ***n* = 40**
Sex, *n* (%)					< 0.001				
Male, *n* = 143	57 (19.1)	46 (15.4)	42 (14.1)	127 (42.6)		86 (28.9)	97 (32.6)	101 (33.9)	16 (5.4)
Female, *n* = 155	30 (10.1)	3 (1.0)	2 (0.7)	131 (44.0)		125 (41.9)	152 (51.0)	153 (51.3)	24 (8.1)
Mean age ± SD (years)	59.4 ± 5.6	59.0 ± 5.6	59.9 ± 6.7	56.2 ± 8.5	< 0.001	54.3 ± 9.9	54.9 ± 9.7	44.5 ± 4.5	51.4 ± 12.7
Age range, *n* (%)									
~ 34	-	-	-	-		2 (0.7)	2 (0.7)	2 (0.7)	2 (0.7)
35–39	-	-	-	-		9 (3.0)	9 (3.0)	9 (3.0)	9 (3.0)
40–44	-	-	-	19 (6.4)		21 (7.0)	21 (7.0)	21 (7.0)	2 (0.7)
45–49	-	-	-	48 (16.1)		52 (17.4)	52 (17.4)	52 (17.4)	4 (1.3)
50–54	25 (8.4)	12 (4.0)	11 (3.7)	52 (17.4)		33 (11.1)	46 (15.4)	47 (15.8)	6 (2.0)
55–59	27 (9.1)	16 (5.4)	15 (5.0)	55 (18.5)		35 (11.7)	46(15.4)	47 (15.8)	7 (2.3)
60–64	15 (5.0)	12 (4.0)	10 (3.4)	35 (11.7)		24 (8.1)	27 (9.1)	29 (9.7)	4 (1.3)
65–69	16 (5.4)	7 (2.3)	6 (2.0)	30 (10.1)		17 (5.7)	26 (8.7)	27 (9.1)	3 (1.0)
70–74	4 (1.3)	2 (0.7)	2 (0.7)	14 (4.7)		11 (3.7)	13 (4.4)	13 (4.4)	1 (0.3)
75–79	0	0	-	5 (1.7)		7 (2.3)	7 (2.3)	7 (2.3)	2 (0.7)
80 ~	0	-	-	0		0	0	0	0
Risk factors, *n* (%)									
Smokers, *n* = 83	47 (56.6)	49 (59.0)	44 (53.0)	82 (98.8)	< 0.001	36 (43.4)	34 (41.0)	39 (47.0)	1 (1.2)
Passive smoking, *n* = 260	78 (30.0)	44 (16.9)	37 (14.2)	251 (96.5)	< 0.001	182 (70.0)	216 (83.1)	223 (85.8)	9 (3.5)
Family history of lung cancer, *n* = 56	40 (71.4)	12 (21.4)	12 (21.4)	49 (87.5)	< 0.001	16 (28.6)	44 (78.6)	44 (78.6)	7 (12.5)
Occupational exposure, *n* = 8	8 (100.0)	5 (62.5)	4 (50.0)	8 (100.0)	0.033	0 (0)	3 (37.5)	4 (50.0)	0 (0)
Having COPD, *n* = 30	25 (83.3)	8 (26.7)	8 (26.7)	27 (90.0)	< 0.001	5 (16.7)	22 (73.3)	22 (73.3)	3 (10.0)

^*^Comparison of those lung cancers deemed eligible according to the four guidelines

### Characteristics of lung cancers confirmed

Additional File: Table S3 shows the stage of diagnosis, and demographic characteristics of lung cancer in this study. In this population, 298 lung cancer patients and 352 lung cancers were pathologically comfirmed, of which the percentage of patients with stage 0 and I lung cancer was 78.1% (275/352). Adenocarcinoma was the most prevalent type of lung cancer at 71.3% (251/352). The highest probability of lung cancer occurred in the age group 50–59. The 10 cases of lung cancer that occurred under 40 years of age in this study were all at stage 0 or I.

## Discussion

In this study, we analyzed the LCS results of 31,394 participants to compare the efficiencies of four domestic and international eligibility criteria for identifying high-risk populations in China and reported the baseline LCS results with LDCT. The results of this study showed that the NCCN guideline was the most efficient of the four LCS guidelines; however, the guideline had the lowest eligibility rate, with an eligibility rate of 6.97%. Moreover, our results revealed that the I-ELCAP eligibility criteria, while capturing the most high-risk populations, had the lowest LCS ERe values among the four guidelines. Relatively relaxed eligibility criteria, despite covering a broad screening population, resulted in extremely low lung cancer detection rates among those who met the criteria, suggesting that guideline eligibility criteria need to be developed in a way that balances the eligibility coverage with lung cancer detection rates. Evaluating the relationship between expanding LC eligibility criteria and increasing the potential risk associated with LCS is an important step to be explored in the future. Despite the CGSL guideline accounting for additional risks, only 29.2% of lung cancer individuals met all eligibility criteria, with the smoking criterion having the lowest compliance rate of the four guidelines. Our study found a statistically significant difference in risk factors among those lung cancer patients who met the eligibility criteria for the four guidelines. A significant proportion of lung cancers did not meet the age eligibility criteria set by the CGSL, NCCN, and USPSTF guidelines, with the highest proportion observed in the 45 − 49 age group. There were sex disparities in eligibility for LCS based on the four guidelines, which may caused by the fact that most women were never smokers and that women had a greater percentage of lung cancers that did not meet eligibility criteria. 78.1% of lung cancers in this LCS were at stage 0 or I and 71.3% were adenocarcinomas.

In another Chinese real-world study that included 15,996 participants with 142 cases of lung cancer, only 9.2% of participants met the USPSTF 2013 criteria [12]. Compared to the results of this study, our results were higher, with approximately 14.8% (44/298) of lung cancer cases meeting the USPSTF 2021 criteria. This is because we used the USPSTF 2021 guidelines, which extended the eligibility criteria for LCS beyond what was outlined in the USPSTF 2013 guidelines, reducing the screening age from 55 to 50 years and lowering the required smoking history from 30 to 20 pack-years. Furthermore, Nemesure et al. [21] found that 49.2% of 1207 pathologically confirmed lung cancer cases met the USPSTF 2013 eligibility criteria, and 41.0% of lung cancer cases met the NCCN criteria, which was consistent with the NLST criteria (age 55–74, smoking history ≥ 30 pack-years, and current tobacco user or quit within the past 15 years). Only 14.8% and 16.4% of the lung cancer cases in our study met the USPSTF 2021 and NCCN 2022 eligibility criteria, respectively. A cross-sectional study demonstrated that 31.1% of the screened population was eligible according to the USPSTF 2021 guidelines [22], whereas only 9.3% of the Chinese population in our real-world study was eligible for USPSTF 2021 guidelines. However, even with the less restrictive USPSTF 2021 eligibility criteria, approximately 85.2% of patients with lung cancer in our cohort did not meet these criteria. These findings are not surprising, as more than 30% of lung cancer cases in Asia are not caused by smoking [23]. This percentage is significantly higher than that in Europe and North America (where the proportion is only 10–15%) [24]. Only 5.2% of females with lung cancers in China are smokers, whereas approximately 84.3% of females with lung cancers in the USA are smokers [25]. In addition, the prevalence of smoking among Chinese men is 52.1%, whereas that among Chinese women is extremely low at 2.7% [26]. The USPSTF and NCCN guidelines that use age and smoking status to define the LCS criteria are not appropriate for the Chinese population, especially for non-smoking women.

Our results showed that the I-ELCAP guideline captured the largest number of screened individuals, with lung cancer detection rates much higher than the other three guidelines, even though the screening efficiency of the I-ELCAP eligibility criteria was the lowest of the four guidelines. This was in line with the purpose of the I-ELCAP guideline, which is to prioritize early diagnosis, and the focus is on detecting lung cancer as early as possible [27]. The I-ELCAP protocol has been adopted by over 80 institutions across 10 countries, forming the I-ELCAP network. One notable aspect of the I-ELCAP guideline is its inclusion of non-smokers in the screening protocol since 2001 [28]. This shows the flexibility of the I-ELCAP protocol, allowing investigator groups to establish their criteria for participation in the screening program [27]. While relatively less stringent eligibility criteria standards may capture a larger number of individuals for screening, it may also result in a higher number of false positives or unnecessary screenings. It is important to consider the trade-off between the number of individuals screened and the efficiency of the screening process. While capturing a larger number of individuals for screening may seem beneficial in terms of potentially detecting more cases of LC, it also increases the burden on healthcare resources and may lead to unnecessary procedures for individuals who do not have LC.

We must recognize that the CGSL guidelines in the current study, despite encompassing selection criteria for occupational exposure, COPD, and a family history of lung cancer, remained unsatisfactory. In the current study population, of the 298 participants diagnosed with lung cancer, 88.6% did not meet the 30-pack-year smoking requirements, and 87.9% did not meet the 20-year passive smoking requirements. These results suggest that the CGSL guideline should consider relaxing the smoking criteria, thereby increasing the number of lung cancer cases eligible for guideline-based screening and achieving the goal of improving LCS efficiency. Approximately 70.8% (211/298) of lung cancer cases in the present study did not meet the CGSL guideline, with women accounting for 80.6%. Thus, significant sex differences exist in the Chinese guidelines for lung cancer diagnosis. Air pollution is an important risk factor for lung cancer in non-smoking women [29]. Previous studies have demonstrated a significant association between fine particles (PM2.5) and an increased risk of lung cancer mortality in China, particularly in women [30]. This may have contributed to the large number of female lung cancer patients who did not meet the Chinese guideline criteria, as air pollution may have played an important role in lung cancer development. However, risk factors associated with lung cancer differ between men and women, such as genetic variations, hormone levels, and carcinogen-associated viruses [31]. Briefly, lung cancer risk factors vary based on sex, indicating that strategies targeting individuals at high risk of LCS should be tailored to each sex.

Our study found that the proportion of lung cancers that did not meet the age criteria of the CGSL, NCCN, and USPSTF guidelines was greater than 30% and that the highest age group for underdiagnosis of lung cancer in the CGSL, NCCN, and USPSTF guidelines was between 45 and 49. Previous studies have also found that among 8392 employees from six hospitals in different Chinese regions, the lung cancer detection rate in the ≤ 40 years age group was 17.3% [20]. These findings suggest that lung cancer can occur in individuals younger than traditionally expected. However, further validation and evaluation are required to determine if these findings can be generalized to other Chinse regions. Moreover, further research is required to examine whether this guideline should be changed to a lower minimum screening age cut-off value of less 50 years.

To improve the eligibility rate and efficiency ratio, CGSL should have more relaxed age and smoking criteria, and NCCN and USPSTF can appropriately consider high-risk factors such as family history of lung cancer, history of occupational exposure, and COPD, and appropriately reduce the number of packs smoked and prolong the number of years of cessation of smoking. I-ELCAP can appropriately increase age thresholds and have stricter smoking criteria. On the one hand, the expansion of eligibility criteria, although allowing more people to be screened may seem beneficial because of the possibility of detecting more lung cancer cases, but on the other hand, it can lead to a series of problems caused by overdiagnosis and overtreatment, such as an increased burden on healthcare resources, invasive tests, radiation damage, and psychological burden on patients. Further research is needed to determine the optimal balance between the efficiency ratio and the overdiagnosis brought about. This will help to develop guidelines that both maximize lung cancer detection and minimize unnecessary screening and false positive results.

Ongoing research efforts are focused on stratifying lung cancer risk to determine the eligibility of individuals best suited for LCS in Asia. Various models have been developed based on sociodemographic characteristics and other risk factors using data from large cohorts [32, 33]. One example is the 2021 lung cancer risk prediction model based on the Korean smoking population, which is more efficient than the LCS guidelines [33]. The model considers specific characteristics of the Korean population and provides a more accurate assessment of lung cancer risk [33]. Similarly, the Chinese NCC-LCm2021 model accurately reflects cancer risk among individuals in the Chinese population, regardless of smoking status [32]. These models highlight the importance of geographically specific risk models. However, the efficiency of these models for assessing screening strategies must be confirmed in a real-world setting. The development of emerging technologies, such as molecular markers, imaging histology, and artificial intelligence (AI), can help optimize LCS strategies. This can help in tailoring screening strategies to high-risk individuals, ensuring that resources are utilized effectively [34]. AI algorithms can integrate clinical, genetic, and demographic data to develop personalized risk prediction models, enabling targeted screening strategies for individuals at higher risk. Moreover, AI can aid in the automation of repetitive tasks, reducing the workload on healthcare professionals and improving efficiency [35]. In summary, by leveraging these emerging technologies, LCS strategies could be optimized in several ways.

The results of our study showed that 78.1% of lung cancers screened by LDCT were at stage 0 or I and 71.3% were adenocarcinomas. In an observational study in Japan, the study population included a total of 12,114 subjects, and 152 cases of lung cancer were diagnosed in 133 participants, of which 85.5% were in clinical stage IA and 88.8% were adenocarcinomas [36]. The results of our study were similar to it. In addition, the study used the NLST criteria of ≥ 30 pack-years of smoking, and approximately 70% of cancer patients could have been missed, further confirming the limitation of using LCS guidelines in Asia, which is dominated by never-smokers with lung cancer [36].

To our knowledge, a comparison of the efficiency of eligibility criteria using these four guidelines in the Chinese population has not been reported in previous studies. We discovered that the screening efficiency was lower, regardless of which guidelines were used. With the exception of the I-ELCAP guidelines, compliance with the screening eligibility criteria and lung cancer detection rates were low. Our findings provide valuable support for future guideline updates and the further refinement of lung cancer risk models.

Our study had some limitations. First, this is a single-center study and, therefore, may have a selection bias that needs to be further validated by multicenter studies. However, on the one hand, because our hospital is the National Cancer Center of China, we were able to attract participants from across China, which to some extent compensated for the shortcomings of a single-center study. On the other hand, including a reported 15,996 participants who completed the baseline LCS at the Health Management Centre of West China Hospital, the results of this study showed that only 9.2% and 24.4%, respectively, met the USPSTF2013 eligibility criteria and Chinese expert consensus. The high missed diagnosis rates for lung cancer of 90.8% and 75.6%, respectively [12], were similar to the results we found in this study. Thus demonstrating that the risk from our selection bias had a negligible effect on the results, we will conduct multicentre studies in the future to further reduce the potential impact of bias. Second, the participants in this study participated in screening voluntarily and were required to pay for the associated costs, leading to a selection bias that may have affected the results. Third, we only collected data on occupational exposure to asbestos and soot; data on occupational exposure to beryllium, uranium, or radon were lacking.

## Conclusions

In conclusion, our results showed that the four guidelines differ in terms of eligibility rates and efficiency ratios, with the highest eligibility rate among the four guidelines being I-ELCAP and the lowest being NCCN. However, the I-ELCAP has the lowest efficiency ratio and the NCCN has a relatively high efficiency ratio for those deemed eligible by the guideline. In the future, the eligibility criteria for the four guidelines will need to be further revised to improve both eligibility rates and efficiency ratios. Further research is required to investigate the eligibility criteria of the guidelines for the Chinese population, particularly sex disparities in LCS eligibility. These findings provide valuable insights for improving LCS guidelines in the Chinese population.

### Supplementary Information

Additional file 1: Table S1. Characteristics of participants based on different guideline eligibility criteria. Table S2. Guideline-based sex stratification of eligibility criteria for lung cancer detected. Table S3. Characteristics of lung cancers confirmed.

## Data Availability

According to the regulations of the Ethics Committee of our hospital, the data generated during the study will be available only after providing the appropriate application.

## Abbreviations

AIS: Adenocarcinoma in situ
CGSL: China guideline for the screening and early detection of lung cancer
COPD: Chronic obstructive pulmonary disease
I-ELCAP: International Early Lung Cancer Action Program
LC: Lung cancer
LCS: Lung cancer screening
LDCT: Low-dose computed tomography
NCCN: National Comprehensive Cancer Network
NLST: National Lung Screening Trial
NSCLC: Non-small cell lung cancer
PACS: Picture archiving and communication system
SI: Smoking index
USPSTF: United States Preventive Services Task Force
