# Supplementary material for: Assessing the efficiency of eligibility criteria for low-dose computed tomography lung screening in China according to current guidelines
Source: BMC Med. 2024 Jun 26;22:267. doi: 10.1186/s12916-024-03445-5 (PMC11210050; doi:10.1186/s12916-024-03445-5)
Supplement: Supplementary file 1 — Additional file 1: Table S1. Characteristics of participants based on different guideline eligibility criteria. Table S2. Guideline-based sex stratification of eligibility criteria for lung cancer detected. Table S3. Characteristics of lung cancers confirmed. [file 12916_2024_3445_MOESM1_ESM.docx]

**Table S1. Characteristics of participants based on different guideline eligibility criteria**

| **Characteristics** | **CGSL eligible, n (%)** | | **Total, n (%)** | **NCCN eligible, n (%)** | | **Total, n (%)** | **USPSTF eligible, n (%)** | | **Total, n (%)** | **I-ELCAP eligible, n (%)** | | **Total, n (%)** | ***P** value** |
| --- | --- | --- | --- | --- | --- | --- | --- | --- | --- | --- | --- | --- | --- |
| Total | Female | Male |  | Female | Male |  | Female | Male |  | Female | Male |  |  |
| Sex | 1921 | 4044 | 5965 | 144 | 2845 | 2989 | 168 | 2751 | 2919 | 9772 | 13139 | 22911 | ＜0.001 |
| Mean age ± SD (years) | 57.7±5.9 | 57.5±5.6 | 57.5±5.7 | 60.0±7.7 | 57.7±6.0 | 57.9±6.1 | 59.3±7.4 | 57.0±5.5 | 57.0±5.7 | 52.1±8.3 | 51.8±8.3 | 51.9±8.0 | ＜0.001 |
| Age range |  |  |  |  |  |  |  |  |  |  |  |  |  |
| ~34 | - | - | - | - | - | - | - | - | - | - | - | - |  |
| 35~39 | - | - | - | - | - | - | - | - | - | - | - | - |  |
| 40-44 | - | - | - | - | - | - | - | - | - | 1920(19.6) | 2834(21.6) | 4754(20.7) |  |
| 45-49 | - | - | - | - | - | - | - | - | - | 2365(24.2) | 3173(24.1) | 5538(24.2) |  |
| 50-54 | 636(33.1) | 1575(38.9) | 2211(37.1) | 49(34.0) | 1030(36.2) | 1079(36.1) | 59(35.1) | 1142(41.5) | 1201(41.1) | 2081(21.3) | 2689(20.5) | 4770(20.8) |  |
| 55-59 | 708(36.9) | 1084(26.8) | 1792(30.0) | 29(20.1) | 876(30.8) | 905(30.3) | 35(20.8) | 849(30.9) | 884(30.3) | 1511(15.5) | 2115(16.1) | 3626(15.8) |  |
| 60-64 | 335(17.4) | 798(19.7) | 1133(19.0) | 27(18.8) | 534(18.8) | 561(18.8) | 32(19.0) | 461(16.8) | 493(16.9) | 1071(11.0) | 1258(9.6) | 2329(10.2) |  |
| 65-69 | 181(9,4) | 446(11.0) | 627(10.5) | 20(13.9) | 274(9.6) | 294(9.8) | 20(11.9) | 222(8.1) | 242(8.3) | 524(5.4) | 669(5.1) | 1193(5.2) |  |
| 70-74 | 61(3.2) | 141(3.5) | 202(3.4) | 13(9.0) | 86(3.0) | 99(3.3) | 16(9.5) | 51(1.9) | 67(2.3) | 190(1.9) | 248(1.9) | 438(1.9) |  |
| 75-79 |  |  |  | 4(2.8) | 33(1.2) | 37(1.2) | 6(3.6) | 26(0.9) | 32(1.1) | 77(0.8) | 107(0.8) | 184(0.8) |  |
| 80~ |  |  |  | 2(1.4) | 12(0.4) | 14(0.5) |  |  |  | 33(0.3) | 46(0.4) | 79(0.3) |  |
| Risk factors |  |  |  |  |  |  |  |  |  |  |  |  |  |
| Smokers, n=9967 | 185(1.9) | 3033(30.4) | 3218(32.3) | 144(1.4) | 2845(28.5) | 2989(30.0) | 168(1.7) | 2751(27.6) | 2919(29.3) | 539(5.4) | 7726(77.5) | 8265(82.9) | ＜0.001 |
| Passive smoking, n=9326 | 243(2.6) | 3388(36.3) | 3631 (38.9) | 134(1.4) | 2634(28.2) | 2768(29.7) | 121(1.3) | 2163(23.2) | 2284(24.5) | 494(5.3) | 8193(87.9) | 8687(93.1) | ＜0.001 |
| Family history of lung cancer, n=5060 | 313(6.2) | 626(12.4) | 939(18.6) | 27(0.5) | 480(9.5) | 507(10.0) | 26(0.5) | 409(8.1) | 435(8.6) | 1815(35.9) | 2004(39.6) | 3819(75.5) | ＜0.001 |
| Occupational exposure, n=1942 | 159(8.2) | 577(29.7) | 736(37.9) | 7(0.4) | 255(13.1) | 262(13.5) | 7(0.4) | 205(10.6) | 212(10.9) | 262(13.5) | 1070(55.1) | 1332(68.6) | ＜0.001 |
| Having COPD, n=3525 | 808(22.9) | 862(24.5) | 1670(47.4) | 21(0.6) | 368(10.4) | 389(11.0) | 19(0.5) | 314(8.9) | 333(9.4) | 1223(34.7) | 1491(42.3) | 2714(77.0) | ＜0.001 |

*Note:* * comparison of those participants deemed eligible according to the four guidelines

**Table S2. Guideline-based sex stratification of eligibility criteria for lung cancer detected**

| **Lung cancer** | **Male, n (%)** | **Female, n (%)** | ***P* value** | **Total, n (%)** |
| --- | --- | --- | --- | --- |
| Total | 143(48.0) | 155(52.0) |  | 298 |
| CGSL eligible | 57(39.9) | 30(19.4) | ＜0.001 | 87( 29.2) |
| CGSL ineligible | 86(60.1) | 125(80.6) |  | 211(70.8) |
| NCCN eligible | 46(32.2) | 3(1.9) | ＜0.001 | 49(16.4) |
| NCCN ineligible | 97(67.8) | 152(98.1) |  | 249(83.6) |
| USPSTF eligible | 42(29.4) | 2(1.3) | ＜0.001 | 44(14.8) |
| USPSTF ineligible | 101(70.6) | 153(98.7) |  | 254 (85.2) |
| I-ELCAP eligible | 127(88.8) | 131(84.5) | 0.310 | 258(86.6) |
| I-ELCAP ineligible | 16(11.2) | 24(15.5) |  | 40(13.4) |

Abbreviations: CGSL = Chinese guidelines for the screening and early detection of lung cancer;

NCCN = National Comprehensive Cancer Network; USPSTF = US Preventive Services Task Force;

I-ELCAP = International Early Lung Cancer Action Program

**Table S3. Characteristics of lung cancers confirmed**

| **Characteristics** | **0, n (%)** | **IA+ IB, n (%)** | **IIA+ IIB, n (%)** | **IIIA+ IIIB, n (%)** | **IV, n (%)** | **Unknown, n (%)** | **Total, n (%)** |
| --- | --- | --- | --- | --- | --- | --- | --- |
| Sex (No. of patients ) | 21(7.0) | 203(68.1) | 19(6.4) | 12(4.0) | 6(2.0) | 37(12.4) | 298(100.0) |
| Male- (143) | 6(28.6) | 89(43.8) | 15(78.9) | 9(75.0) | 3(50.0) | 21(56.8) | 143(48.0) |
| Female-(155) | 15(71.4) | 114(56.2) | 4(21.1) | 3(25.0) | 3(50.0) | 16(43.2) | 155(52.0) |
| Histologic type |  |  |  |  |  |  |  |
| Total (No. of lesions) | 43(12.2.) | 232(65.9) | 19(5.4) | 14(4.0) | 7(2.0) | 37(10.5) | 352(100.0) |
| AIS | 43(12.2) | 0 | 0 | 0 | 0 | 0 | 43(12.2) |
| Adenocarcinoma | 0 | 217(61.6.) | 15(4.3) | 10(2.8) | 7(2.0) | 2(0.6) | 251(71.3) |
| Mucinous adenocarcinoma | 0 | 11(3.1) | 0 | 0 | 0 | 0 | 11(3.1) |
| Squamous cell carcinoma | 0 | 3(0.9) | 0 | 3(0.9) | 0 | 1(0.3) | 7(2.0) |
| NSCLC | 0 | 0 | 0 | 0 | 0 | 1(0.3) | 1(0.3) |
| Small cell lung cancer | 0 | 0 | 2(0.6) | 1(0.3) | 0 | 2(0.6) | 5(1.4) |
| Other types* | 0 | 1(0.3) | 2(0.6) | 0 | 0 | 3(0.9) | 6(1.7) |
| N/A | - | - | - | - | - | 28(8.0) | 28(8.0) |
| Age range |  |  |  |  |  |  |  |
| Total (No.of patients ) | 21(7.0) | 203(68.1) | 19(6.4) | 12(4.0) | 6(2.0) | 37(12.4) | 298(100.0) |
| ~39 | 1(4.8) | 8(3.9) | 1(5.3) | 0 | 0 | 0 | 10(3.4) |
| 40-49 | 7(33.3) | 49(24.1) | 4(21.1) | 2(16.7) | 1(16.7) | 8(21.6) | 71(23.8) |
| 50-59 | 8(38.1) | 85(41.9) | 6(31.6) | 4(33.3) | 1(16.7) | 17(45.9) | 121(40.6) |
| 60-69 | 5(23.8) | 50(24.6) | 4(21.1) | 4(33.3) | 2(33.3) | 9(24.3) | 74(24.8) |
| 70-79 | 0 | 11(5.3) | 4(21.1) | 2(16.7) | 2(33.3) | 3(8.1) | 22(7.4) |

*Note:* Other types*: IA+ IB: 1 carcinoid, IIA+ IIB:1 basal cell carcinoma, 1 adenosquamous; Unknown: 1 lymphoma, 2 metastases

Abbreviations: AIS: adenocarcinoma in situ, N/A*: data not shown because patients were transferred to other hospitals, NSCLC: non-small cell lung cancer.
